# Determinants of Clinical Cure and Mortality in Patients With Stenotrophomonas maltophilia Infections: A Retrospective Analysis

**DOI:** 10.7759/cureus.86294

**Published:** 2025-06-18

**Authors:** Silvia Costa, Francisca Bartilotti Matos, Ana Gorgulho, Celina Gonçalves, Cristóvão Figueiredo, Daniel Coutinho, Tiago Teixeira, Margarida Pargana, Gabriela Abreu, Luís Malheiro

**Affiliations:** 1 Medicine, Faculdade de Medicina da Universidade do Porto, Porto, PRT; 2 Infectious Diseases, Unidade Local de Saúde Gaia Espinho, Vila Nova de Gaia, PRT; 3 Microbiology, Unidade Local de Saúde Gaia Espinho, Vila Nova de Gaia, PRT; 4 Infectious Diseases, Unidade Local de Saúde Gaia Espinho, Vila Nova De Gaia, PRT; 5 Health Sciences, Universidade de Aveiro, Aveiro, PRT

**Keywords:** critical care outcomes, infection control, multi-drug resistance, nosocomial infection, stenotrophomonas maltophilia infection

## Abstract

Introduction

*Stenotrophomonas maltophilia *(*S. maltophilia*)is an emerging opportunistic Gram-negative bacillus associated with high morbidity and mortality. However, data on its epidemiology in Portugal are limited. This study evaluated the clinical characteristics, risk factors, and outcomes of patients with* S. maltophilia *colonization and infection.

Methods

This retrospective, single-center cohort study was conducted on adult patients with *S. maltophilia* isolated from clinical specimens between 2020 and 2024. Patients were classified as infected or colonized, with infected cases further divided into survivors and non-survivors. Clinical, microbiological, and treatment data were analyzed using univariate and multivariate methods to identify risk factors for 30-day mortality.

Results

Among 97 patients, 62.9% had *S. maltophilia* infection and 37.1% were colonized. Infected patients more frequently had cardiovascular disease (31.1% vs. 13.9%) and immunosuppression (24.6% vs. 11.1%). Targeted antimicrobial therapy was administered to 78.7% of infected patients, primarily with co-trimoxazole. Mortality was higher in infected patients (32.8% vs 8.3%, p=0.003). Non-survivors had significantly higher rates of septic shock (66.7% vs. 12.2%, p < 0.01), recent antibiotic exposure (100.0% vs. 73.2%, p = 0.011), and cardiovascular disease (55.0% vs. 19.5%, p = 0.08). Multivariate analysis identified a lack of appropriate therapy (odds ratio (OR) 20.78, 95% CI 1.35-318.6), cardiovascular disease (OR 11.85, 95% CI 1.39-100.49), and septic shock (OR 13.18, 95% CI 1.10-157.37) as independent predictors of 30-day mortality.

Conclusion

*S. maltophilia* infections, especially in critically ill patients, are associated with high mortality, especially in patients with septic shock, recent antibiotic exposure, and cardiovascular disease. Lack of appropriate antibiotics is also an independent risk factor for mortality. Early diagnosis, prompt targeted therapy, and optimal management of comorbidities are crucial to improving outcomes.

## Introduction

*Stenotrophomonas maltophilia* (*S. maltophilia*) is an emerging opportunistic Gram-negative bacillus. Although not intrinsically virulent, it exhibits multidrug resistance against many classes of antibiotics, including beta (β)-lactams, quinolones, aminoglycosides, and tetracyclines [1-3]. These intrinsic resistance mechanisms are further worsened by additional factors, including reduced outer membrane permeability, multidrug efflux pumps, and chromosomally encoded beta (β)-lactamases such as OXA-48 [3,4].

The incidence of *S. maltophilia* infections is increasing in both nosocomial and community settings worldwide [3]. Transmission primarily occurs through direct patient-to-patient contact, indirect contact via fomites (such as contaminated surfaces, medical equipment, or healthcare professionals’ hands), or through aerosolization during the use of contaminated nebulizers [4]. Outbreaks in intensive care units (ICUs) are common and are typically associated with contaminated environmental wet sources and cross-transmission from healthcare professionals or medical devices to patients [5,6]. A major clinical challenge is that *S. maltophilia* readily colonizes the respiratory tract of patients with chronic lung disease or those receiving mechanical ventilation and forms persistent biofilms on implanted devices, making it difficult to differentiate harmless colonization from true infection [1,3,7].

Although infections by *S. maltophilia* are uncommon when compared to colonization, it is one of the 10 most common microorganisms associated with ICU-acquired pneumonia in Europe [8]. As *S. maltophilia* is associated with complex clinical infections, with attributable mortality rates as high as 37.5%, these infections should not be overlooked [9]. Furthermore, treatment has become increasingly challenging due to the rising incidence of acquired resistance to trimethoprim-sulfamethoxazole (co-trimoxazole), the first-line therapy, and the uncertain efficacy of alternative treatments [3]. 

The incidence and clinical impact of *S. maltophilia* colonization and infection are largely unknown in Portugal. Outcomes and risk factors for infection and antimicrobial susceptibility testing are usually inferred from case series in other countries [10]. Understanding local epidemiology is essential for developing evidence-based recommendations to prevent colonization and improve infection management. This study, therefore, aimed to evaluate the demographic and clinical characteristics of patients with *S. maltophilia* colonization and infection, with a focus on factors associated with clinical cure and mortality.

## Materials and methods

Study design and setting

This retrospective, single-center cohort study was conducted at the tertiary care university hospital Unidade Local de Saúde de Gaia Espinho (ULSGE), Vila Nova de Gaia, Portugal. Ethical approval was obtained from the institutional ethics committee, and the requirement for informed consent was waived due to the retrospective design (approval number: 183/2024).

Study population

This study included patients with *S. maltophilia* isolated from biological samples between January 1, 2020, and May 1, 2024. Cases were identified using institutional microbiological surveillance software. The inclusion criteria were as follows: (1) isolation of *S. maltophilia* in a biological sample, (2) age ≥18 years, and (3) availability of complete medical records, defined as comprehensive clinical documentation containing sufficient information to distinguish colonization from infection, assess comorbidities, and evaluate relevant clinical outcomes. Only the first isolate was considered a case, and repeated isolates were excluded. Cases were excluded if a clear distinction between colonization and infection was not possible.

Data collection and management

Cases were identified with the institutional microbiology surveillance system. Data were extracted from electronic medical records by trained researchers according to a standardized protocol to ensure consistency and reliability. *S. maltophilia* isolates were initially cultured on appropriate agar media and subsequently identified using matrix-assisted laser desorption ionization-time of flight (MALDI-TOF) mass spectrometry. Following European Committee on Antimicrobial Susceptibility Testing (EUCAST) recommendations [11], antimicrobial susceptibility testing (AST) for co-trimoxazole was performed through the disk diffusion method or minimum inhibitory concentration (MIC) determination, using the gradient strip method E-test® (bioMérieux, Craponne, France) or turbidimetry (VITEK2®, bioMérieux).

Infection and colonization were distinguished based on clinical presentation and the need for treatment. Colonization was defined as the isolation of *S. maltophilia* from biological samples in the absence of clinical signs or symptoms of infection. Infection was defined as the isolation of *S. maltophilia* from an appropriate biological sample collected in response to a new clinical sign or symptom (such as clinical deterioration) suggestive of infection, with antimicrobial therapy either initiated or deemed warranted. When the distinction between colonization and infection was not possible or was uncertain, three reviewers independently evaluated the registries, and a decision was reached only when all three agreed.

The demographic variables included patient age and sex. The clinical variables included patient location (ward, ICU, outpatient), reason for admission (surgical, medical, infection-related), antimicrobials administered in the last 90 days, presence of invasive medical devices or prostheses, previous colonization by *S. maltophilia*, and comorbidities such as chronic pulmonary disease, diabetes mellitus, cardiovascular disease, and immunosuppression. Immunosuppression was defined as the presence of HIV infection with a CD4+ count <200 cells/µL, active hematologic malignancy, solid organ or stem cell transplantation, ongoing chemotherapy or radiotherapy, chronic use of immunosuppressive therapy (including corticosteroids ≥20 mg/day for ≥14 days, calcineurin inhibitors, antimetabolites, mTOR inhibitors, or biologic agents), or a diagnosed primary immunodeficiency. The severity of illness at the time of diagnosis was assessed and classified into three categories: no sepsis, sepsis, or septic shock, according to the SEPSIS-3 recommendations [12]. Microbiological data included the microbiological sample in which *S. maltophilia* was isolated, the time from admission to symptom onset or biological sample collection, the time from sample collection to *S. maltophilia* identification, and the antimicrobial susceptibility profile of the isolates.

The therapeutic variables documented included the empirical antimicrobial therapy administered before culture results, the appropriateness of this therapy based on susceptibility testing and antimicrobial dosing, the subsequent definitive therapy, and the time from symptom onset or sample collection to the initiation of appropriate antimicrobials. For the purposes of this study, appropriate dosing was defined as co-trimoxazole at 10-15 mg/kg/day of the trimethoprim component or levofloxacin at 750 mg once daily or 500 mg twice daily. Modifications to the initial treatment regimens were noted, including adjustments made after culture results, reasons for switching, use of combination antibiotic regimens, and duration of therapy. The outcomes included 30-day mortality, admission to the hospital if the initial diagnosis was made in the outpatient setting, length of hospital stay (measured in days), need for admission to an intensive care unit, and infection recurrence in the following 90 days. Infection recurrence was defined as a new isolation of *S. maltophilia* in a biological sample, accompanied by clinical signs or symptoms of infection, after an initial course of appropriate antimicrobials accompanied by clinical improvement.

Sample size and statistical analysis

As all eligible cases were included, no sample size calculations were performed. Descriptive statistics were used to summarize the baseline characteristics. Continuous variables were described as mean and standard deviation (SD) if the distribution was normal and median and interquartile range (IQR) if the distribution was not normal. In the first analysis, colonized patients were compared with infected patients. In the second analysis, only infected patients were considered, and survivors were compared with non-survivors. Comparisons were performed using chi-square tests for categorical variables and Student’s t-tests or the Mann-Whitney U test for continuous variables. Multivariate logistic regression was used to identify the risk factors for clinical cure and mortality. Results are reported as odds ratios (OR) with 95% confidence intervals (CI). Data analyses were performed using IBM SPSS Statistics software, version 27.0 (IBM Corp., Armonk, NY, USA)

## Results

Patient population and baseline characteristics

Initially, 119 cases with *S. maltophilia* isolates were identified; 22 duplicate cases were removed, leaving 97 patients for analysis. Among these, 61 (62.9%) had an *S. maltophilia* infection, and 36 (37.1%) were colonized (Table 1). No patients were excluded based on inability to distinguish infection from colonization. The mean ages were similar (64.9 ± 13.7 years for infected vs. 63.4 ± 14.3 years for colonized, p=0.773), and a higher proportion of infected patients were male (38, 62.3% for infected vs. 16, 44.0% for colonized, p=0.087).

**Table 1:** Baseline characteristics and outcomes of patients with documented Stenotrophomonas maltophilia infection and colonization COPD: chronic obstructive pulmonary disease; ICU: intensive care unit; IQR: interquartile range; n: number; NA: not applicable; SD: standard deviation

Variables	Infection (n = 61)	Colonization (n = 36)
Demographics		
Age (years), mean (SD)	64.9 (13.7)	63.4 (14.3)
Male sex, n (%)	38 (62.3)	16 (44.0)
Female sex, n (%)	23 (37.7)	20 (55.6)
Patient location		
Hospital ward, n (%)	21 (34.4)	13 (36.1)
Hospital ICU, n (%)	24 (39.3)	9 (25.0)
Outpatient, n (%)	16 (26.2)	14 (38.9)
Reason for admission		
Medical, n (%)	13 (21.3)	6 (16.7)
Surgical, n (%)	32 (52.5)	16 (44.4)
Chronic lung disease		
Any chronic lung disease, n (%)	20 (32.8)	15 (41.5)
COPD, n (%)	12 (19.6)	9 (25.0)
Bronchiectasis, n (%)	9 (14.8)	8 (22.2)
Interstitial lung disease, n (%)	2 (3.2)	0
Other comorbidities		
Diabetes mellitus, n (%)	21 (34.4)	14 (38.9)
Cardiac disease, n (%)	19 (31.1)	5 (13.9)
Oncological disease, n (%)	14 (23.0)	8 (22.2)
Immunosuppression, n (%)	15 (24.6)	4 (11.1)
Invasive medical devices		
Any invasive device, n (%)	31 (50.8)	16 (44.4)
Urinary catheter, n (%)	17 (27.9)	10 (27.8)
Central venous catheter, n (%)	20 (32.8)	6 (16.7)
Orotracheal tube, n (%)	15 (24.6)	4 (11.1)
Tracheostomy, n (%)	4 (6.6)	5 (13.9)
Intravascular or articular prosthesis, n (%)	8 (13.1)	1 (2.8)
External ventricular drainage, n (%)	2 (3.3)	1 (2.8)
Nasogastric tube, n (%)	16 (26.2)	7 (19.4)
Other, n (%)	5 (8.2)	5 (13.9)
Outcomes		
Sepsis or septic shock, n (%)	23 (37.7)	NA
Length of stay (days), median (IQR)	50.0 (37.5)	15.0 (35.0)
Admission to the hospital if diagnosed as an outpatient, n (%)	7 (11.5)	1 (2.8)
30-day mortality, n (%)	20 (32.8)	3 (8.3)
Infection recurrence, n (%)	3 (4.9)	NA

Patient location differed slightly: similar proportions were admitted to hospital wards (21, 34.4% for infected vs. 13, 36.1% for colonized, p=0.867), while ICU admissions were more common among infected patients (24, 39.3% for infected vs. nine, 25.0% for colonized, p=0.156). In contrast, colonized patients were more frequently managed as outpatients (16, 26.2% for infected vs. 14, 38.9% for colonized, p=0.168).

Comorbidities such as cardiac disease (19, 31.1% for infected vs. five, 13.9% for colonized, p=0.057) and immunosuppression (15, 24.6% for infected vs. four, 11.1% for colonized, p=0.106) were more prevalent in the infection group, whereas chronic lung disease (20, 32.8% for infected vs. 15, 41.5% for colonized, p=0.379) was slightly higher in colonized patients. Invasive devices were more frequent in infected patients (31, 50.8% for infected vs. 16, 44.4% for colonized), with central venous catheters noted in 20 (32.8%) infected cases. Notably, previous colonization with *S. maltophilia* was observed only in infected patients (five, 8.2%). Recent antibiotic exposure was slightly higher among infected patients (50, 82.0% for infected vs. 26, 72.2% for colonized, p=0.260), and for those hospitalized, the interval from admission to *S. maltophilia* detection was comparable (18.5 ± 24.5 vs. 21.0 ± 23.0 days, p=0.437).

Microbiological findings and treatment

Microbiological and treatment descriptions can be found in Table 2. Recent antibiotic exposure was slightly higher among infected patients (50, 82.0% for infected vs. 26, 72.2% for colonized, p=0.260), and for those hospitalized, the interval from admission to *S. maltophilia* detection was comparable (18.5 ± 24.5 vs. 21.0 ± 23.0 days, p=0.437). Respiratory samples were the predominant source of isolation (40, 65.6% for infection vs. 22, 61.1% for colonization). Isolates from biopsies, pus, or exudates were more common in the infection group (17, 27.9% for infection vs. six, 16.7% for colonization), while blood isolates were rare (two, 3.3% for infection vs. two, 5.6% for colonization). Urine samples yielded *S. maltophilia* more frequently among colonized patients (one, 1.6% for infection vs. four, 11.1% for colonization). The time to microorganism identification was similar between groups (4.0 ± 3.0 for infection vs. 4.2 ± 2.1 days for colonization, p=0.568). Susceptibility testing showed that most isolates were susceptible with augmented exposure to co-trimoxazole (56, 91.8% for infection vs. 35, 97.2% for colonization), with resistance observed in three (4.9%) and one (2.8%) cases, respectively. Co-infections were identified in 41 (67.2%) infected patients and 19 (52.8%) colonized patients. Targeted therapy was administered in 48 (78.7%) infected patients compared to three (8.3%) colonized cases. Adverse effects attributed to co-trimoxazole were observed in seven patients (mental confusion, vomiting, rash, hypervolemia, acute kidney injury, thrombocytopenia, and swallowing difficulties), leading to a switch to ciprofloxacin in two cases.

**Table 2:** Microbiological and treatment description of patients with documented Stenotrophomonas maltophilia infection and colonization. IQR: interquartile range; n: number; NA: not applicable; SD: standard deviation

Variables	Infection (n = 61)	Colonization (n = 36)
Previous colonization with *S. maltophilia*, n (%)	5 (8.2)	0
Exposure to antibiotic therapy in the previous 90 days, n (%)	50 (82.0)	26 (72.2)
Recent hospitalizations or healthcare exposures in the past 90 days, n (%)	19 (31.1)	10 (27.8)
Time since hospital admission and *S. maltophilia* isolation (days), median (IQR)	18.5 (24.5)	21.0 (23.0)
Time to microorganism identification (days), median (IQR)	4.0 (3.0)	4.2 (2.1)
Type of sample from which *S. maltophilia* was isolated		
Blood, n (%)	2 (3.3)	2 (5.6)
Respiratory, n (%)	40 (65.6)	22 (61.1)
Urine, n (%)	1 (1.6)	4 (11.1)
Biopsy/pus/exudate, n (%)	17 (27.9)	6 (16.7)
Cerebrospinal fluid, n (%)	1 (1.6)	1 (2.8)
Biliary fluid, n (%)	1 (1.6)	0
Peritoneal fluid, n (%)	0	1 (2.8)
Co-trimoxazole susceptibility profile		
Susceptible with augmented exposure, n (%)	56 (91.8)	35 (97.2)
Resistant, n (%)	3 (4.9)	1 (2.8)
Not tested, n (%)	2 (3.3)	0
Co-infections with another microorganism in the same sample		
Any microorganism, n (%)	41 (67.2)	19 (52.8)
Bacteria, n (%)	38 (62.3)	19 (52.8)
Viruses, n (%)	2 (3.3)	0
Fungi, n (%)	6 (9.8)	2 (5.6)
Treatment description		
Time to anti-stenotrophomonas antimicrobial (days), median (IQR)	6.0 (11.0)	4.0 (0.8)
Received appropriate treatment for *S. maltophilia*, n (%)	48 (78.7)	3 (8.3)
Treatment duration (days), median (IQR)	14.0 (10.25)	8 (10)

Outcomes

Sepsis or septic shock was present in 23 (37.7%) infected patients, and the 30-day mortality rate was significantly higher in the infection group (20, 32.8% vs. 3, 8.3%, p=0.003). Infected patients also had a longer median hospital stay (50.0 days (IQR: 37.5) vs. 15.0 days (IQR: 35.0), p=0.013) (Table 1). Among initially outpatient cases, seven (11.5%) in the infection group required subsequent hospital admission, and infection recurrence was documented in three (4.9%) of the infected patients.

Comparison of survivors and non-survivors

Table 3 presents a detailed comparison between survivors (n=41) and non-survivors (n=20). Non-survivors were significantly more likely to require ICU admission (14, 70.0% of non-survivors vs. 10, 24.4% of survivors, p=0.006) and were less often managed as outpatients (one, 5.0% of non-survivors vs. 15, 36.6% of survivors, p=0.006). Chronic lung disease was more prevalent among survivors (17, 41.5% of survivors vs. three, 15.0% of non-survivors, p=0.046), while recent antibiotic exposure was universal in non-survivors (20, 100.0% of non-survivors vs. 30, 73.2% of survivors, p=0.011). The median time to identification was shorter in non-survivors (3.0 vs. 5.0 days, p=0.023), and septic shock was markedly more common (12, 66.7% of non-survivors vs. five, 12.2% of survivors, p<0.01). Additionally, appropriate anti-*S. maltophilia* therapy was less frequently administered in non-survivors (11, 55.5% of non-survivors vs. 37, 90.2% of survivors, p=0.003). Survivors experienced longer treatment durations (14.0 vs. 8.0 days) and hospital stays (38.0 vs. 22.0 days, p=0.047). Almost none of the patients in either group received appropriate empirical antimicrobials (most received meropenem or piperacillin/tazobactam ± vancomycin, according to institutional protocols). In the multivariate analysis, the lack of appropriate treatment for *S. maltophilia* (OR 20.78, 95% CI 1.35-318.6), underlying cardiovascular disease (OR 11.85, 95% CI 1.39-100.49), and the presence of septic shock (OR 13.18, 95% CI 1.10-157.37) were independently associated with 30-day mortality.

**Table 3:** Clinical and demographic characteristics of survivors and non-survivors *S. maltophilia*: *Stenotrophomonas maltophilia*; ICU: intensive care unit; IQR: interquartile range; n: number; NA: not applicable; SD: standard deviation

Variables	Survivors (n = 41)	Non-survivors (n = 20)	p-value
Demographics			
Age, mean (sd)	65.1 (11.1)	64.5 (18.2)	0.118
Male sex, n (%)	24 (58.5)	14 (70.0)	0.417
Female sex, n (%)	17 (41.5)	6 (30.0)	
Patient location			
Hospital ward, n (%)	16 (39.0)	5 (25.0)	0.180
Hospital ICU, n (%)	10 (24.4)	14 (70.0)	0.006
Outpatient, n (%)	15 (36.6)	1 (5.0)	0.006
Comorbidities			
Chronic lung disease, n (%)	17 (41.5)	3 (15.0)	0.046
Diabetes mellitus, n (%)	13 (31.7)	8 (40.0)	0.574
Cardiac disease, n (%)	8 (19.5)	11 (55.0)	0.08
Oncological disease, n (%)	8 (19.5)	6 (30.0)	0.517
Immunosuppression, n (%)	10 (24.4)	5 (25.0)	1.000
Other characteristics			
Invasive medical devices, n (%)	17 (41.5)	14 (70.0)	0.056
Previous colonization with *S. maltophilia*, n (%)	4 (9.8)	1 (5.0)	1.000
Exposure to antibiotic therapy in the previous 90 days, n (%)	30 (73.2)	20 (100.0)	0.011
Recent hospitalizations or healthcare exposures in the past 90 days, n (%)	13 (31.7)	6 (30.0)	1.000
Time since hospital admission and infection (days), median (IQR)	16.0 (15.0)	18.0 (17.0)	0.278
Time to microorganism identification (days), median (IQR)	5.0 (5.0)	3 (1.75)	0.023
Co-infections in the same site, n (%)	29 (70.7)	12 (60.0)	0.562
Co-trimoxazole susceptibility profile			
Susceptible with augmented exposure, n (%)	38 (97.2)	18 (90.0)	0.546
Resistant, n (%)	3 (7.3)	0	
Primary site of infection			
Blood, n (%)	1 (2.4)	1 (5.0)	0.990
Respiratory, n (%)	24 (58.5)	15 (75.0)	0.746
Urinary, n (%)	0 (0.0)	1 (5.0)	1.000
Skin and soft tissue, n (%)	4 (9.8)	1 (5.0)	0.442
Osteoarticular, n (%)	11 (26.8)	0 (0.0)	0.999
Central nervous system, n (%)	0 (0.0)	1 (5.0)	1.000
Intrabdominal, n (%)	1 (2.4)	1 (5.0)	1.000
Severity of infection			
No sepsis, n (%)	34 (82.9)	4 (22.2)	<0.01
Sepsis	2 (4.9)	2 (11.1)	0.058
Septic shock, n (%)	5 (12.2)	12 (66.7)	<0.01
Treatment characteristics			
Adequate empirical treatment, n (%)	2 (3.3)	0	1.000
Directed anti-*S. maltophilia* treatment, n (%)	37 (90.2)	11 (55.5)	0.003
Levofloxacin, n (%)	7 (17.1)	0	0.084
Co-trimoxazole, n (%)	31 (75.6)	11 (55.5)	0.142
Combination therapy, n (%)	2 (3.3)	0	1.000
Surgical source control, n (%)	13 (10.8)	3 (5.2)	0.222
Time to anti-*S. maltophilia* treatment (days), median (IQR)	7.0 (13.0)	5.0 (5.0)	0.223
Treatment duration (days), median (IQR)	14.0 (11.0)	8.0 (19.0)	0.175
Length of stay (days), median (IQR)	38.0 (46.0)	22.0 (33.0)	0.047

## Discussion

This study provides essential insights into the clinical characteristics, risk factors, and outcomes associated with *S. maltophilia *colonization and infection. To our knowledge, this is the first comprehensive case series conducted in Portugal, offering a detailed perspective on the local epidemiology and risk factors. It is also one of the few studies that differentiates and compares colonization and infection by this microorganism.

Most patients in our cohort were of advanced age and had multiple comorbidities, particularly in the infection group, suggesting an association between *S. maltophilia* and frailty. Colonization and infection were observed across all clinical settings; however, infection was more common in the ICU, whereas colonization was more frequently identified in outpatient settings, further suggesting that critical illness is a risk factor for *S. maltophilia* infection. Additionally, surgery appeared to be associated with *S. maltophilia* infection and colonization, as more than two-thirds of patients with *S. maltophilia* identification had undergone surgical procedures.

During the study period, over one-third of *S. maltophilia* identifications were classified as colonization cases. Distinguishing *S. maltophilia* infection from simple colonization is challenging because the organism is common in hospital water systems and readily forms biofilms on airways and devices; thus, it is often recovered from tracheal aspirates, urine, or wounds in patients who have fever, leukocytosis, or infiltrates for other reasons [13]. Consequently, respiratory tract isolates more often represent colonization rather than infection, which aligns with our finding that the respiratory tract was the most frequent colonization site [13]. In the ICU, these nonspecific clinical signs and the absence of reliable quantitative cutoffs in routine cultures mean that a positive culture may reflect carriage rather than disease.

Clinical indicators, imaging, and laboratory data are crucial for the diagnosis of infection. However, while *S. maltophilia* identification in sterile sites such as blood or cerebrospinal fluid typically suggests a true infection in the appropriate clinical context, these findings should be interpreted with caution because in up to half of the cases they may result from contamination, potentially leading to unnecessary antimicrobial use [14]. More than half of the colonized patients had another microorganism isolated in the same biological sample. Coinfection has been described as an advantage for *S. maltophilia*, as it may promote biofilm production, further complicating the distinction between infection and colonization [1, 4, 15]. Additionally, invasive devices, such as central venous catheters and external ventricular drains, may become colonized, especially after prolonged hospitalization, making diagnosis more difficult, even when *S. maltophilia *is isolated from a sterile site.

Practical ways to improve accuracy may include using a composite definition that links clinical deterioration with repeated high-density isolates, requesting quantitative or protected-specimen cultures for respiratory samples, tracking host-response biomarkers such as procalcitonin to gauge true infection, and discussing equivocal cases in a joint meeting of infectious-disease, microbiology, and critical-care teams. Adopting such safeguards may reduce overtreatment and make future studies more comparable across centers.

Rønn et al. [16] demonstrated that *S. maltophilia* detection in patients with chronic obstructive pulmonary disease (COPD) correlates with higher mortality and hospitalization rates over five years. In contrast, our cohort showed low 30-day mortality and 90-day hospital admission rates among colonized patients, likely due to the shorter follow-up period that may have overlooked later complications.

We observed high rates of antimicrobial exposure in the 90 days prior to *S. maltophilia* identification, suggesting that prior antibiotic use may contribute to colonization or infection, possibly by altering the microbiome and selecting for resistant organisms [17]. Similar findings have been reported in patients with COPD and ICU-acquired pneumonia [18, 19]. The most frequently administered antibiotics included penicillins, carbapenems, and cephalosporins, with similar usage patterns in both colonized and infected patients.

Among infected patients, the 30-day mortality rate was notably high. Patients initially diagnosed in the outpatient setting had higher rates of hospital admission, and among survivors, hospital stays were significantly longer. These findings suggest a worse prognosis for those who develop infections. Additionally, the time to microorganism identification was significantly shorter in non-survivors, possibly reflecting higher bacterial loads, a feature rarely described in other studies. Mortality rates as high as 56% have been reported for patients with *S. maltophilia* infections [20, 21]. Two recent meta-analyses focusing on different populations with *S. maltophilia* infection identified risk factors for mortality similar to those found in our study, reinforcing that comorbidities, critical illness, and invasive devices are key contributors to infection-related mortality [22]. Because *S. maltophilia* disproportionately affects frail, critically ill patients, who experience the highest mortality, there is an urgent need for robust evidence to guide optimal management.

As observed in other studies, polymicrobial infections were common among both survivors and non-survivors of *S. maltophilia* infections [20]. This raises the question, as suggested by other authors, of whether the high mortality rate truly reflects *S. maltophilia* infection or is overestimated, as co-infecting microorganisms may be more invasive, making it difficult to directly attribute deaths to *S. maltophilia* infection [23]. Given that most empirical antibiotic regimens did not cover *S. maltophilia* in either survivors or non-survivors, early diagnosis is crucial for guiding appropriate antimicrobial therapy and reducing mortality.

However, the existing literature presents conflicting findings regarding the impact of appropriate therapy on mortality. While some studies have found no significant difference in mortality between patients who received appropriate therapy and those who did not, others, including the present study, suggest that targeted antibacterial therapy is significantly associated with lower mortality. A meta-analysis demonstrated that patients who received inappropriate antimicrobial therapy had significantly higher mortality [20].

Treatment options are limited, with co-trimoxazole being the first-line therapy for *S. maltophilia* infections. However, its use may be restricted because of its adverse effects. Despite concerns regarding the rising co-trimoxazole resistance [24, 25], our study found that resistance was relatively uncommon. Levofloxacin is a potential alternative, with some evidence suggesting non-inferiority to co-trimoxazole while exhibiting similar resistance rates [24, 25]. Further challenges include the absence of EUCAST-defined MIC breakpoints for alternative agents such as fluoroquinolones, minocycline, aztreonam/avibactam, and cefiderocol [11], which limits treatment standardization and therapeutic decision-making. However, MIC breakpoints for some of these agents may be available in the Clinical & Laboratory Standards Institute (CLSI) guidelines, which can assist in guiding therapy when EUCAST criteria are lacking [26]. Additionally, studies evaluating the optimal duration of therapy and directly comparing the efficacy of different antimicrobials for *S. maltophilia* infections are lacking, further complicating the development of evidence-based treatment recommendations. Clinical trials comparing the effectiveness of commonly used agents are scarce. Although the Infectious Diseases Society of America (IDSA) guidelines provide recommendations on antibiotic choices and support combination therapy, they acknowledge that the quality of evidence is low and that a definitive “standard of care” regimen against which to evaluate alternative treatments has not been established [27]. Nevertheless, emerging agents like cefiderocol and aztreonam-avibactam are gaining traction as credible alternatives to co-trimoxazole, supported by an expanding body of evidence [28, 29].

This study had several strengths. This is one of the first comprehensive analyses of *S. maltophilia* infection and colonization in Portugal, providing valuable epidemiological data on this emerging pathogen. The distinction between colonization and infection allows for a more accurate assessment of the risk factors and outcomes. Additionally, the inclusion of a diverse patient population across multiple settings, including ICU, inpatient, and outpatient cases, enhances the generalizability of our findings. A detailed analysis of antimicrobial exposure, co-infections, and treatment regimens further strengthens our conclusions regarding risk factors and the impact of appropriate therapy on patient outcomes.

However, as a single-center, retrospective study, the findings may not be fully generalizable to other healthcare settings with different patient populations, infection control practices, or antimicrobial stewardship policies. The relatively small sample size may have reduced our ability to detect certain associations, particularly in the subgroup analyses. We found that infected patients were more common than colonized patients, which is surprising, given that *S. maltophilia* is generally considered a microorganism of low virulence [13]. However, this may represent a reporting bias, as the microbiology laboratory may not have identified *S. maltophilia* in unsuitable samples such as urine and sputum considered to have a polymicrobial flora with likely contamination. Furthermore, treatment decisions were made at the discretion of the attending physicians, leading to variations in antibiotic selection, duration, and management strategies, which may have influenced patient outcomes. Detailed data regarding the type of surgical procedures, such as whether they were elective or emergency interventions or whether the surgical site involved sterile or infected fields, were not consistently available in the retrospective records and could not be reliably incorporated into the analysis, representing a limitation of the study. Finally, the absence of long-term follow-up data restricted the ability to evaluate infection recurrence and long-term patient outcomes. Future research should focus on larger multicenter prospective studies to validate these findings, explore alternative treatments, define optimal treatment durations, and improve antimicrobial stewardship strategies for *S. maltophilia* infections.

## Conclusions

*S. maltophilia* poses a substantial clinical burden, particularly in critically ill or frail patients, among whom we observed a high 30-day mortality. Early, accurate differentiation between colonization and true infection, prompt initiation of adequate antimicrobials, and vigilance for coinfections are essential to improve outcomes and uphold antimicrobial-stewardship principles. Although high-dose trimethoprim-sulfamethoxazole remains the first-line option, our findings highlight the need for prospective studies to determine optimal treatment duration, assess emerging agents, and develop targeted prevention strategies for high-risk populations.

## References

[1] Looney WJ, Narita M, Mühlemann K (2009). Stenotrophomonas maltophilia: an emerging opportunist human pathogen. Lancet Infect Dis.

[2] Trifonova A, Strateva T (2019). Stenotrophomonas maltophilia - a low-grade pathogen with numerous virulence factors. Infect Dis (Lond).

[3] Falagas ME, Valkimadi PE, Huang YT, Matthaiou DK, Hsueh PR (2008). Therapeutic options for Stenotrophomonas maltophilia infections beyond co-trimoxazole: a systematic review. J Antimicrob Chemother.

[4] Brooke JS (2012). Stenotrophomonas maltophilia: an emerging global opportunistic pathogen. Clin Microbiol Rev.

[5] Matheu MM, Rice N, Singson K (2022). Stenotrophomonas maltophilia outbreak associated with sink drains in an intensive care unit. Open Forum Infect Dis.

[6] Gideskog M, Welander J, Melhus Å (2020). Cluster of S. maltophilia among patients with respiratory tract infections at an intensive care unit. Infect Prev Pract.

[7] Majumdar R, Karthikeyan H, Senthilnathan V, Sugumar S (2022). Review on Stenotrophomonas maltophilia: an emerging multidrug‐resistant opportunistic pathogen. Recent Pat Biotechnol.

[8] (2025). Healthcare-associated infections acquired in intensive care units - Annual Epidemiological Report for 2021. https://www.ecdc.europa.eu/en/publications-data/healthcare-associated-infections-acquired-intensive-care-units-annual-0.

[9] Falagas ME, Kastoris AC, Vouloumanou EK, Rafailidis PI, Kapaskelis AM, Dimopoulos G (2009). Attributable mortality of Stenotrophomonas maltophilia infections: a systematic review of the literature. Future Microbiol.

[10] Banar M, Sattari-Maraji A, Bayatinejad G (2023). Global prevalence and antibiotic resistance in clinical isolates of Stenotrophomonas maltophilia: a systematic review and meta-analysis. Front Med (Lausanne).

[11] (2025). European Committee on Antimicrobial Susceptibility Testing: clinical breakpoints - breakpoints and guidance. http://www.eucast.org/clinical_breakpoints.

[12] Singer M, Deutschman CS, Seymour CW (2016). The Third International Consensus Definitions for sepsis and septic shock (Sepsis-3). JAMA.

[13] Bhaumik R, Aungkur NZ, Anderson GG (2023). A guide to Stenotrophomonas maltophilia virulence capabilities, as we currently understand them. Front Cell Infect Microbiol.

[14] Aysert-Yıldız P, Yıldız Y, Habibi H, Eser S, Özgen-Top Ö, Özger HS, Dizbay M (2022). Stenotrophomonas maltophilia bacteremia: from diagnosis to treatment. Infect Dis Clin Microbiol.

[15] McDaniel MS, Lindgren NR, Billiot CE, Valladares KN, Sumpter NA, Swords WE (2023). Pseudomonas aeruginosa promotes persistence of Stenotrophomonas maltophilia via increased adherence to depolarized respiratory epithelium. Microbiol Spectr.

[16] Rønn C, Kamstrup P, Eklöf J (2023). Mortality and exacerbations associated with Stenotrophomonas maltophilia in chronic obstructive pulmonary disease. A regional cohort study of 22,689 outpatients. Respir Res.

[17] Aitken SL, Sahasrabhojane PV, Kontoyiannis DP (2021). Alterations of the oral microbiome and cumulative carbapenem exposure are associated with Stenotrophomonas maltophilia infection in patients with acute myeloid leukemia receiving chemotherapy. Clin Infect Dis.

[18] Soumagne T, Levesque F, Milot J, Godbout K, Lacasse Y, Maltais F (2021). Significance of Stenotrophomonas maltophilia when detected in sputum of ambulatory patients with COPD. Int J Chron Obstruct Pulmon Dis.

[19] Wang N, Tang C, Wang L (2021). Risk factors for acquired Stenotrophomonas maltophilia pneumonia in intensive care unit: a systematic review and meta‐analysis. Front Med (Lausanne).

[20] Huang C, Lin L, Kuo S (2024). Risk factors for mortality in Stenotrophomonas maltophilia bacteremia - a meta-analysis. Infect Dis (Lond).

[21] Hasbek M, Aldemir Ö, Çakır Kıymaz Y, Baysal C, Yıldırım D, Büyüktuna SA (2025). Mortality rates and risk factors associated with mortality in patients with Stenotrophomonas maltophilia primary bacteraemia and pneumonia. Diagn Microbiol Infect Dis.

[22] Garazi M, Singer C, Tai J, Ginocchio CC (2012). Bloodstream infections caused by Stenotrophomonas maltophilia: a seven-year review. J Hosp Infect.

[23] Kannangara D, Pandya D (2020). Stenotrophomonas maltophilia: attributable mortality may be overestimated. Int J Infect Dis.

[24] Watson L, Priestley L, Chapman SJ, Andersson MI, Jeffery K, Flight WG (2022). Clinical associations with carriage of pulmonary Stenotrophomonas maltophilia. J R Coll Physicians Edinb.

[25] Bostanghadiri N, Sholeh M, Navidifar T, Dadgar-Zankbar L, Elahi Z, van Belkum A, Darban-Sarokhalil D (2024). Global mapping of antibiotic resistance rates among clinical isolates of Stenotrophomonas maltophilia: a systematic review and meta-analysis. Ann Clin Microbiol Antimicrob.

[26] Clinical and Laboratory Standards Institute (2024). CLSI M100: Performance Standards for Antimicrobial Susceptibility Testing. https://clsi.org/shop/standards/m100/.

[27] Tamma PD, Heil EL, Justo JA, Mathers AJ, Satlin MJ, Bonomo RA (2024). Infectious Diseases Society of America 2024 guidance on the treatment of antimicrobial-resistant Gram-negative infections. Clin Infect Dis.

[28] Soueges S, Faure E, Parize P (2025). Real-world multicentre study of cefiderocol treatment of immunocompromised patients with infections caused by multidrug-resistant Gram-negative bacteria: CEFI-ID. J Infect.

[29] Wong-So J, Magréault S, Carbonnelle É, Jullien V, Desgrouas M, Boulain T, Barbier F (2025). Aztreonam plus ceftazidime-avibactam for post-neurosurgical meningitis due to Stenotrophomonas maltophilia. Antimicrob Agents Chemother.

